# Current perspectives between metabolic syndrome and cancer

**DOI:** 10.18632/oncotarget.8341

**Published:** 2016-03-24

**Authors:** Carla Micucci, Debora Valli, Giulia Matacchione, Alfonso Catalano

**Affiliations:** ^1^ Department of Clinical and Molecular Sciences, Polytechnic University of Marche, School of Medicine, Ancona, Italy

**Keywords:** metabolic syndrome, cancer risk, visceral adiposity, hyperglycemia, inflammation

## Abstract

Metabolic syndrome is a cluster of risk factors that lead to cardiovascular morbidity and mortality. Recent studies linked metabolic syndrome and several types of cancer. Although metabolic syndrome may not necessarily cause cancer, it is linked to poorer cancer outcomes including increased risk of recurrence and overall mortality. This review tends to discuss the major biological and physiological alterations involved in the increase of incidence and mortality of cancer patients affected by metabolic syndrome. We focus on metabolic syndrome-associated visceral adiposity, hyperinsulinemia, hyperglycemia, insulin-like growth factor (IGF-I) pathway as well as estrogen signaling and inflammation. Several of these factors are also involved in carcinogenesis and cancer progression. A better understanding of the link between metabolic syndrome and cancer may provide new insight about oncogenesis. Moreover, prevention of metabolic syndrome &ndash; related alterations may be an important aspect in the management of cancer patients during simultaneous palliative care.

## INTRODUCTION

Metabolic syndrome (MetS) is increasing in incidence and lead to significance cardiovascular disease (CVD) and mortality. CVD includes all the disease of the heart and circulation including coronary heart disease, angina, heart attach and stroke. MetS can also raise the risk of other diseases, including cancer. It's thought that more than 2 in 10 cancers in the UK are linked to being MetS. The relationship between MetS and cancer is complex. Individual components of the metabolic syndrome are known as risk factors for incident cancer disease, but it is not clear how the clustering of these components is linked to the development and progression of tumors. It seems self-evident that a condition characterized by multiple risk factors, as the metabolic syndrome, will carry a greater risk for adverse clinical outcomes than will a single risk factor. Therefore, a better understanding of the relationship between components of the metabolic syndrome and whether and how these components contribute to progression of cancer and its incidence could inform more effective prevention strategies [1].

MetS rises with economic development, sedentary lifestyle and associated overweight and obesity as seen among populations in Asia, South and North America, and Eastern Europe. As a result, the metabolic syndrome is now both a public health and a clinical problem. MetS has existed in various forms and definitions [2]; however the most widely accepted definition was issued by the Adult Treatment Panel III of the National Cholesterol Education Program (NCEP-ATP III). According to the NCEP-ATPIII definition, MetS is defined having three or more of the following five risk factors: 1) visceral obesity defined by waist circumference (population and country specific definitions); 2) triglycerides ≥ 150 mg/dL; 3) low high-density lipoprotein (HDL) cholesterol levels (men ≤ 40 mg/dL; women ≤ 50 mg/dL); 4) blood pressure ≥ 130 and/or 85 mmHg; and 5) fasting glucose ≥ 100 mg/dL [3].

The third National Health and Nutrition Examination Survey (NHANES III) criteria have shown that about 47 million people have MetS [4]. Incidence increases with age and it has been estimated that, in the category over 50 years of age, MetS affects more than 40% of the population in the United States and nearly 30% in Europe [5, 6]. The reasons for this incidence vary from person to person. It can sometimes be linked to genes we were born with, or our environments, as well as our individual behaviour and choices. And some drugs and diseases can also contribute to weight gain.

Interestingly patients with MetS are at twice the risk of developing CVD over the next 5 to 10 years as individuals without the syndrome, whereas it has been shown that metabolic syndrome confers a 5-fold increase in risk for type 2 diabetes [3]. On the other hand, research has shown that many types of cancer are more common in people who have MetS, such as breast cancer, in women after the menopause, bowel cancer, colon cancer, esophageal cancer, gastric cancer, pancreatic, kidney and liver cancer. This is probably due to harmful effects in the body that can have MetS, like producing hormones and growth factors that affect the way our cells work. This review presents current perspectives on the relations of metabolic syndrome with cancer risk, offering new insights into potential biological mechanisms, and suggesting some directions for future cancer treatment.

## METABOLIC SYNDROME AND CANCER

Recently, Esposito *et. al* analyzed 38,940 patients affected by cancer and MetS through a meta-analysis and it has been shown that the MetS is associated with an increased risk of several cancers including colorectal, pancreas and liver cancers. However, many of the reported associations might differ between sexes. In men, MetS was strongly associated with liver (RR 1.43, *P* < 0.0001) and colorectal (RR 1.25, *P* < 0.001) cancers and weakly associated with bladder cancer (RR 1.10, *P* = 0.013). While in women, the presence of metabolic syndrome was associated with endometrial (RR 1.61, *P* = 0.001), pancreas (RR 1.58, *P* < 0.0001), breast (in particular in postmenopausal, RR 1.56, *P* = 0.017), colorectal (RR 1.34, *P* = 0.006) and ovary cancers (RR 1.26, *P* = 0.054) [7].

The increasing prevalence of MetS worldwide and the high incidence of some malignancies, imply that every year many cases of cancer are attributable to metabolic syndrome. Primary prevention and early detection of cancer are recommended for patients affected by fully developed diseases.

**Figure 1:**
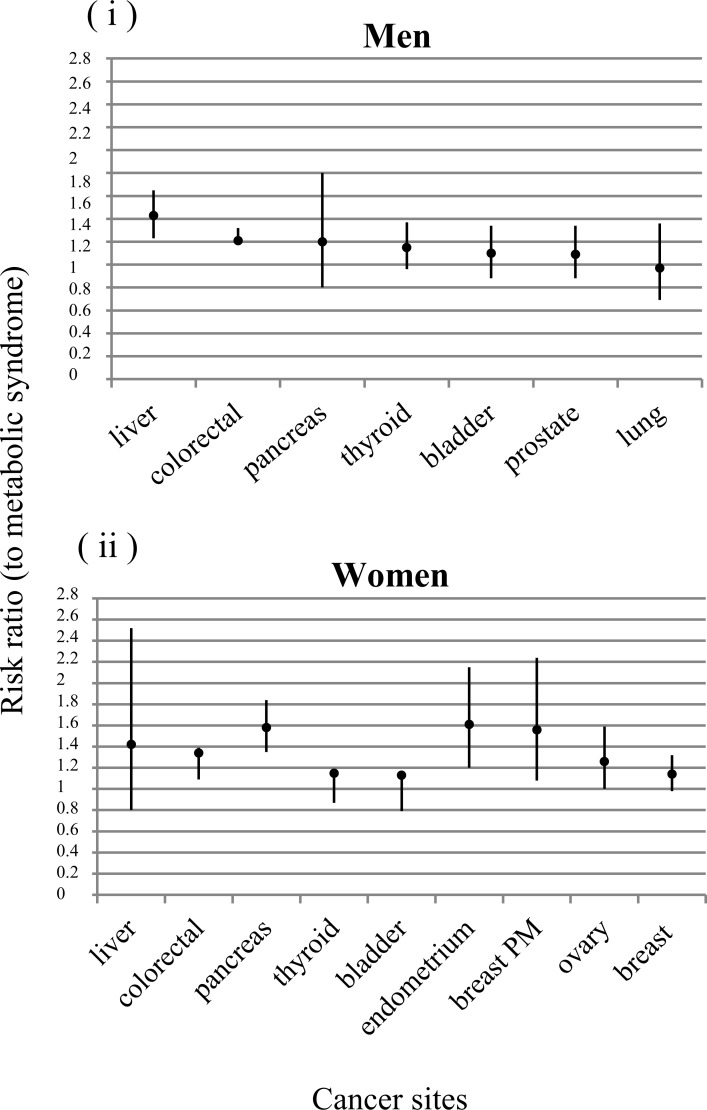
Association between metabolic syndrome and cancer risk Risk ratio in different cancer sites both in men **i.** and in women **ii.**

It's important to underline how interventions to reduce the prevalence of metabolic syndrome in adult populations will reduce cancer risk [8] therefore patients with the metabolic syndrome, even in absence of obesity or diabetes, should be encouraged to undergo appropriate cancer screenings, at least for some more frequently involved sites [9] (Figure 1).

Jaggers *et al.* have conducted a study to examine the association between MetS and all-cause cancer mortality, in which participants were only men enrolled in Aerobics Center Longitudinal study (ACLS) (33,230 aged 20-88 years) who, at the time of examination, were free of known cancer. Using criteria of the NCEP-ATP III, men have been divided into two groups according to have or not MetS. The study has shown that men with MetS had a 56% higher risk of cancer mortality compared with those with only one condition. Moreover, participants with 3 or more risk factors had an 83% higher risk of cancer death compared to men without risk factors. With the exception of high blood pressure, the only component that did not increase cancer risk, it has been shown a positive association between cancer mortality and each of the MetS components. The presence of MetS was then significantly associated with increased risk of cancer mortality for lung and colorectal cancer.

Features studies must be done to analyze the connection between MetS and all cause cancer mortality among female population, although previous studies have shown lower risk of cancer mortality for woman with MetS [10]. Recently Stebbing *et al.* reported through prospective cohort study that woman affected by breast cancer and MetS are non-responders to standard treatment than those without MetS. So preventing or controlling the risk factors of MetS would be one of the possible ways to reduce cancer deaths in both sexes [11].

MetS can also represent a common long-term complication after cancer treatment that affects life expectancy and quality of life. For example, in childhood sarcoma survivors who received chemotherapy, the prevalence of the metabolic syndrome was 33% compared with data in healthy population [12]; for adult survivors of testicular cancer the prevalence of metabolic syndrome was higher in those patients treated with chemotherapy (26%) and surgery only (36%) compared with healthy controls (9%) [13]. Finally, patients with prostate cancer receiving androgen-deprivation therapy had a higher prevalence of MetS (55%) than patients treated with prostatectomy, radiotherapy, or both (22%) and healthy controls (20%) [14]. The presence of MetS in cancer survivors is associated with signs of early atherosclerosis and may represent the connection between cancer treatment and its severe late effects like cardiovascular disease [15].

## MECHANISMS THAT INCREASE THE RISK OF CANCER IN METABOLIC SYNDROME

Patients affected by MetS present several biological and physiological alterations which may increase risk of neoplastic transformation or increase progression of existing cancer.

**Figure 2:**
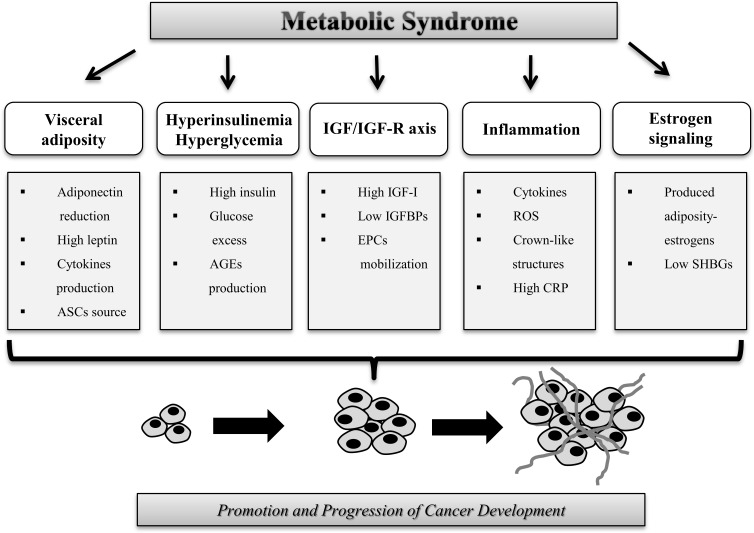
Mechanisms that increase the risk of cancer in patients with metabolic syndrome Biological alterations associated with MetS that influence cancer development and progression such as visceral adiposity, hyperinsulinemia, IGF/IGF-R axis, inflammation and estrogen signaling.

We desire to summarize the main aspects that link MetS and risk of cancer (Figure 2).

### Visceral adiposity

The high rates of obesity are a worldwide problem: the International Obesity Taskforce estimates that 1.1 billion people are overweight (BMI, body mass index, of 25-29.9 kg/m^2^) and 312 million are obese (BMI ≥30 kg/m^2^) [16].

There are two categories of adipose tissue: brown adipose tissue (BAT) and white adipose tissue (WAT), the last one is also divided in subcutaneous and visceral. The visceral-adiposity stores energy as triglycerides and protects organs from mechanical stress. Several epidemiological studies confirmed the relationship between visceral adiposity and an increased risk of developing certain types of cancer [17–19] including colorectal, breast (especially postmenopausal women), endometrial, esophageal adenocarcinoma, cholangiocarcinoma and gastric cardia cancers. The American Cancer Society calculated that currently new cancer cases are in order of 1.5 million with half a million cancer deaths per year, nearly one in five due to obesity [20, 21].

WAT is an active endocrine organ secreting local and systemic hormones (such as leptin and adiponectin), cytokines (such as TNF-α and iterleukin-6) interacting with the immune system and various growth factors: insulin-like growth factor (IGF-1), insulin-like growth factor-binding protein (IGFBPs) and transforming growth factor (TGF-β) [22].

Adipokines (hormones, cytokines and other proteins with signaling properties) are synthesized by adipocytes and regulate many physiological processes, in particular, appetite, angiogenesis, metabolism of glucose and fatty acids, as well as inflammatory and immune reactions [23].

Adiponectin is the most abundant hormone in circulation (0.05% of serum proteins) secreted after activation of the nuclear receptor Peroxisome Proliferator-Activated Receptor-γ (PPAR-γ) in fat cells. It enhances metabolism of glucose and fatty acids (reduction of FFA concentration) in liver and muscle and increasing insulin sensitivity [24, 25]. Adiponectin also has an anti-inflammatory character and it's a negative regulator of angiogenesis, so it is considered to have anticancer effect [26].

A low level of adiponectin is due to adipose tissue hypoxia and this is correlated to a higher risk to develop breast, endometrial and gastric cancers [27].

Leptin is known as the hormone that reduces food intake giving a feeling of satiety [28, 29] as increases insulin sensitivity and lipolysis in adipose tissue The major source of leptin is WAT, thus obese patients become hyperleptinemic for the development of leptin resistance and more susceptible to the risk factors of metabolic syndrome [24]. It was observed that leptin may promote neoplastic transformation, proliferation of cancer cells and tumor angiogenesis [30], indeed high levels of leptin in plasma are associated with prostate, colon, breast and endometrial cancer patients.

Adipocytes, in addiction, secretes proangiogenic factors; among them Vascular Endothelial Growth Factor (VEGF), is one of the most important. VEGF is stimulated by hypoxia and implicated in angiogenesis, fundamental for tumor formation and metastasis [24]. One recent study on obese patients demonstrated an increase in serum VEGF and soluble VEGFR-2 [31], positively dependent on accumulation of visceral adiposity [32].

WAT could be a source of mobilizable progenitor cells [33]: adipose stem cells (ASC) that are WAT-derived mesenchymal stem cells, with potential to differentiate into osteocytes, chondrocytes and adipocytes, may be a possible link between obesity and cancer [34–36]. ASCs can move in response to tumor signal like hypoxia and inflammation and can be use for tumor vasculogenesis. Subsequently the recruitment by tumors, they can be integrated in tumor stroma after transition into cancer associated fibroblasts (CAFs).

Several studies have shown a higher mobilization and recruitment of ASCs in obese patients that lead to stimulation of tumor growth, promotion of angiogenesis and increasing of cancer cells invasion. The great number of circulating ASCs, differentiates in perivascular cells that provide oxygen and nutrient to tumor, inducing an augment survival and limit apoptosis of cancer cells.

Vincenzo Eterno *et al.* have analyzed the role of ASCs in breast recurrence, after surgery, in patients who undergoing autologous fat graft for breast reconstruction and have shown that ASCs are tumorigenic in presence of breast cancer cells which express the tyrosine-kinase receptor c-Met. Moreover the co-injection of ASCs and breast cancer cells in nude mice produces a tumor more vascularizated and increased in size [37].

### Hyperinsulinemia and hyperglycemia

Insulin is the most potent anabolic hormone, secreted by the pancreatic β-cells located in the islets of Langherans. It has a significant role in glucose, fat and protein metabolism [38]. Insulin enables liver cells, muscle and adipose tissue to extract glucose from the bloodstream and it increases glycogen synthesis in muscle and liver cells, esterification of free fatty acids in adipocytes, inhibits lipolysis and gluconeogenesis; stimulates also cell growth and differentiation [39, 40].

In healthy individuals, blood glucose concentrations are maintained through a state of balance between insulin production and insulin-mediated glucose uptake in target tissues [41] determinate by glucose transporters. Insulin resistance can be defined as a condition in which the normal cellular response to insulin is reduced. The pancreatic β-cells react by secreting more insulin, leading to increased circulating insulin concentrations (hyperinsulinemia) to maintain normal plasma glucose concentrations [24]. A favorable niche for neoplastic tissue survival and cancer stem cells development is created by insulin resistance [42–44], through the abnormally high levels of growth factors, adipokines, reactive oxygen species, adhesion factors, and pro-inflammatory cytokines observed under this condition. Chronic hyperinsulinemia is also associated with various types of cancer such as colorectal, pancreatic, endometrial and breast cancer [45], because it reduces the production of insulin-like growth factor-binding protein IGFBP -I and -II, proteins that normally bind to insulin-like growth factor IGF -1 and inhibits its action. These leads to an increase of circulating IGF-I and promotes tumor development through changes in the cellular environment [46].

Metabolic syndrome is also characterized by increased circulating glucose (hyperglycemia). Glucose excess can be converted to macromolecular precursors such as acetyl-CoA for fatty acids, glycolytic intermediates for nonessential amino acids, and ribose for nucleotides [47]. Considering that cancer cells require a lot of energy and substrates to maintain their intensive, uncontrolled proliferation, those cells have an enhanced ability to take up and use glucose. In virtue of this, glucose transporter proteins especially GLUT1 and GLUT3 [25, 48, 49], and enzymes involved in glycolysis such as hexokinase-2 (HK2) have activity and/or expression increased in many tumors.

Furthermore certain types of cancer have been associated with some Tricarboxylic Acid Cycle (TCA) enzymes mutations, including isocitrate dehydrogenase (IDH1 and IDH2) [50], succinate dehydrogenase (SDH) and fumarate hydratase (FH) [51, 52].

Malignant tumor growth is supported also by altered activity of several glycolytic enzymes such as the overexpression of hexokinase [53] and 6-phosphofructo-2-kinase/fructose-2,6- bisphosphatase-4 (PFKFB-4) [54] that enhanced the flux through glycolysis.

Several studies of patients with different tumor types have confirmed that increased glucose uptake/accumulation by tumors, correlates with a higher grade of tumor, incremented metastatic potential, reduced response to therapy and poorer survival. Data showed a statistically significant increase in risk of pancreas cancer, malignant melanoma, and urinary tract cancers among subjects who had elevated levels of fasting glucose. The relationship of hyperglycemia with the risk of cancer overall and of cancer at organ specific sites was emphasized by Stattin *et al.* in a prospective study [55].

Recently our group showed that the hyperglycemic state is sufficient to accelerate lung cancer development in an oncogene K-Ras mouse model.

Indeed, K-Ras-driven tumors exposed to hyperglycemia *in vivo*, grew faster than euglycemic hosts and showed a more malignant growth behavior. Moreover, our current study provides compelling evidence that hyperglycemia, after activation of oncogenic K-Ras, exerts its pro-tumorigenic effects by maintaining a sub-population of cancer tumor-initiating cells, namely lung bronchio-alveolar stem cells (BASCs) [56].

Various signaling pathways that cooperate to control cancer cell behavior are activated by high glucose. Indeed several studies suggest that high glucose induces cancer cell invasiveness and migration through stimulation of epithelial-mesenchymal transition (EMT), a complex process critical for the acquisition of migration, invasiveness and pluripotent stem cell-like phenotype [57].

Recently, Dong *et al.* [58] suggested that the EMT phenotype and the expression of cancer stem cell markers in basal luminal breast carcinoma are hyperglycemia-induced; these conditions lead to reduce the generation of reactive oxygen species (ROS) and to increase cell survival. Hyperglycemia is also an important contributing factor to support rapid proliferation [59].

So these data further support the hypothesis that tumor-promoting activity of hyperglycemia can be associated with several aspects of oncogenesis.

### IGF-I pathway

The IGF system is a complex molecular network that includes two ligands (IGF-I and IGF-II), two receptors (IGF-IR and IGF-IIR), six high-affinity-binding proteins (IGFBP-I-IGFBP-VI) and several binding-protein proteases [60, 61]. IGF-I expression is regulated by insulin and growth hormone (GH) which stimulated the production of IGF-I in liver, the main source of circulating IGF-I. Diet, nutrition, age and sex affect levels of circulating IGF-I and IGFBP-III. IGF-I stimulates cell proliferation and inhibits apoptosis, interacting with its specific receptor on cell membrane, IGF-IR, and with insulin receptor (IR) even if with low affinity [62]. These interactions are regulated by IGFBPs. The IGFBPs can promote stabilization in the circulation, regulation of the efflux from liver to target tissues and availability of IGF-I for binding to its receptors and particularly most of the circulating IGF-I (80%) is bound to IGFBP-III [46].

IGF-I binding to IGF-IR activates two main signaling pathways: phosphatidyloinositol 3-kinase (PI3K)-AKT/protein kinase B (PKB) pathway and the Ras-mitogen-activated protein kinase (MAPK) pathway. Stimulation of PI3K pathway leads to activation of several downstream substrates, including PKB. Its active form (Akt/PKB) enhances proliferation, tumorigenesis and self-renewal by activating mammalian target of rapamycin (mTOR) and forkhead box O (FoxO), and blocking glycogen synthase kinase 3β (GSK3β) that result in accumulation of β-Catenin and in activation of its downstream targets [41].

The same effects are also achieved through the activation of Ras/MAPK/extracellular signal-related kinase 1/2 (ERK-1/2) (21).

Cancer cells show significant overexpression of IGF-I and its receptor. High circulating levels of IGF-I, are associated with increased risk for several cancers, including breast [63], prostate [64], lung [65], and colorectum [66]. Instead the level of IGFBP-III, which suppresses the mitogenic action of IGF-I, is inversely associated with risk of these cancers.

The involvement of IGF-I in cancer progression is supported by several clinical and experimental studies. A significantly increased risk for prostate cancer development is due to an augment of circulating IGF-I as shown by Price et al. (2012) [67], others studies also revealed a specifically expression of IGF-I in tumor tissue in prostate cancer suggesting that levels of IGF-I may be a prognostic marker in predicting risk of death in men with advanced prostate cancer [68–70].

In vitro studies on human colon cancer cells showed cells proliferation promotion by IGF-I, an overexpression of IGF-IR, and inhibition of tumor cell growth using its monoclonal antibody [71]; moreover IGF-I serum levels are increased in patients with locally advanced colorectal cancer (pT3 and pT4), in comparison to less advanced (pT2) [72].

In familial breast cancer an association between high IGF-I levels and cancer development has been proved [73–75] and in breast cancer survivors IGF-I can also predict higher risk of recurrence [76].

Regarded cancer metastasis recently has been documented a role for the IGF system in several human cancer such as colorectal [77] and gastric cancer [78].

IGF-IR is also expressed by endothelial progenitor cells from bone marrow (EPCs). BM-derived cells are precursors for both hematopoietic and endothelial cells; in particular EPCs represent the non-hematopoietic (CD45-) BM derived cell population [79].

Exciting new data have shown that tumor neovascularization, which supports growth and dissemination of tumors, involves recruitment of EPCs. An increased mobilization of EPCs has been associated with cancer, vascular injury, and poor prognosis in patients with lymphoma, thus establishing the significance of these cells in tumor progression.

BM-derived cells are thought to merge with the wall of a growing blood vessel, where they differentiate into endothelial cells [80]. After treatment with vascular-targeting therapies, the number of EPCs increases, and they invade and colonize the viable rim of tumor that remains, thereby contributing to the rapid regrowth [81].

Recently some insights have been obtained about the role of IGF in progenitor cells relocalization, suggesting a role of IGFs during BM-derived cell mobilization.

IGF has an important role in the angiogenic processes, indeed tumor neovasculature is also influenced by IGF which promoting proliferation and migration of endothelial cells, mobilization and colonization of tumor niche by BM-derived cells.

### Inflammation

MetS is frequently associated with inflammation. Regarding hyperglycemia is well-known that an excess of glucose promotes formation and accumulation of advanced glycation end-products (AGEs) [23]. AGEs bind to AGE receptors on macrophages, endothelial and mesangial cells, causing receptor-induced Reactive Oxygen Species (ROS) production. ROS can damage DNA through different mechanisms such as DNA deletions, modifications and frame shifts [82]. DNA damage can affect genes linked to cell survival or cell proliferation like p53 and Ras respectively, and triggers cancer progression. So these compounds cause degenerative changes in cells, alter signaling pathways of their metabolism and may lead to carcinogenic mutations.

Even inflamed adipose tissue may play a critical role in pathogenesis of several cancers, such as breast, colon, pancreas, and kidney [83].

Visceral adipose tissue can release several cytokines as tumor necrosis factor (TNF-α) and interleukin-6 (IL-6) which are considered to form a link between inflammation and cancer. Indeed it has been shown, in obese women, elevated circulating levels of TNF-α and IL-6 which are associated with development and progression of breast tumors [84]. These cytokines are known to promote angiogenesis and they are positively correlated with insulin resistance.

Particularly TNF-α activates two pathways: MAPK and NF-kB pathway. NF-kB is a transcription factor that activates the expression of genes which promote cell proliferation, inhibit apoptosis and therefore enhance cell survival. NF-kB also increases production of nitrogen oxide (NO) and favors formation of ROS [85].

Another family of small cytokines is chemokines, of which the circulating Monocyte Chemoattractant Protein-1 (MCP-1) promotes the recruitment of monocytes to adipose tissue, where the cells differentiate and become macrophages [86].

Infiltrated macrophages surround the adipocyte in a histologically characteristic pattern known as crown-like structures (CLS) [87] and effectively these inflammatory foci were first observed in visceral fat of metabolic syndrome patients.

Moreover, components of metabolic syndrome have a positive correlation with C reactive-protein (CRP), an acute phase protein synthesized and secreted by the liver [85].

Particularly it has been shown a highly significant correlation between visceral adiposity and CRP, and also patients with increasing number of metabolic syndrome components presented a linear increase in CRP levels [89]. This protein is also associated with an augmented risk to developed many types of cancer such as colorectal, cervical and ovarian cancer.

Hence CRP can probably be used as a marker of chronic inflammation in metabolic syndrome patients.

### Estrogen signaling

Visceral adiposity regulates the synthesis of the endogenous sex steroids such as estrogens, androgens and progesterone through several mechanisms. In particular in men and postmenopausal women, adipose tissue is the principal site of estrogens synthesis [46].

In fertile women estrogens, of which oestradiol is the major, are predominantly produced by the ovary. Whereas in menopause, estrogens production decreases and remains a peripheral conversion, primarily in the adipose tissue, of androgens by the cytochrome P450 enzyme aromatase located in adipocytes [86]. As a result, increasing adiposity with age has been suggested to contribute to increase total and free circulating estrogen levels [90].

Another consequence of increased visceral adiposity is reduction in hepatic synthesis and blood concentrations of sex-hormone binding globulin (SHBG), a plasmatic binding protein with high specific affinity for estradiol [91] that generally brings out an increase in the fraction of bioavailable estradiol.

Epidemiological studies have given several evidence that this shift in circulating levels of sex steroids, induced through adiposity, could in large part explain the associations between anthropometric indices of excess weight and risks of breast (postmenopausal women only) and endometrial (both pre- and postmenopausal women) cancers. Especially estrogens show a central role in regulating cellular differentiation, proliferation and apoptosis induction [92–94] in these tissue types, as indicated by a large amount of experimental and clinical demonstrations.

Indeed in estrogen receptor-positive breast and endometrial cancers, estradiol acts as a powerful growth factor that supports tumor growth; estrogen activity through different and complex mechanisms may promote tumor development and progression.

Direct effects of estrogens include stimulation of cellular proliferation and inhibition of apoptosis *via* ER-α agonism as well as induction of vascular endothelial growth factor and angiogenesis [95, 96]. Furthermore, carcinogenesis is probably due to mutagenic effects of estrogen *via* genotoxic metabolites [95].

Differential effects of menopause on cancer incidence observed in epidemiologic studies point to the potential role of estrogen in development and progression of these malignancies.

In postmenopausal women risk of estrogen receptors-positive breast cancer development is inversely related to blood levels of SHBG [97, 98], reply to endometrial cancer in which was reported an increased cancer risk among both pre and postmenopausal women who have comparatively low plasma levels of SHBG [99, 100].

## NEW THERAPEUTIC OPPORTUNITIES

Many therapeutic approaches are studied to face the metabolic syndromes and its impact on development and progression of certain types of cancer.

As described above, insulin is the most important hormone in the metabolic syndrome and its binding with receptors induces inhibition of apoptosis and promotes cell proliferation. Cancer cells are characterized by an overexpression of insulin receptors (IGF-R) suggesting their important role in tumorigenesis and growth. In addition, surrounding stromal tissue of tumor cells produces IGF-I and IGF-II [101] suggesting that activation of the IGF-IRs of tumor cells may be mediated by IGFs in a paracrine and autocrine way [102]. Only recently tools for targeting the IGF pathways are becoming available for therapy. More than 10 IGF/IGF-IR inhibitors have entered clinical trials and they can be divided in three main classes: monoclonal antibodies against IGF-IR; monoclonal antibodies against IGF-I and IGF-II ligands; and IGF-IR tyrosine kinase inhibitors [103]. These molecules, used in clinical trials of patients with tumors, including non-small cell lung cancer, breast cancer, and pancreatic cancer, failed to show clinical benefit. Possible reasons for failure include the complexity of the IGF-IR/insulin receptor system, in fact the IGF-IR can cross-talk with other receptor tyrosine kinase and their downstream effectors and this situation can compensate the inhibition of IGF-IR by a specific antibody. Moreover, the formation of complexes between IGF-IR and specific antibodies lead to an increase of soluble free IGF-I and IGF-II that can leave the circulation to stimulate IGF receptors present on cell surface of cancer cells [104]. Up to date it is still necessary to make a successful IGF-IR target therapy.

Another condition of metabolic syndrome associated with multiple cancers is obesity. This metabolic condition is characterized by the deregulation of adipokines such as leptin and adiponectin responsible of maintenance of metabolic homeostasis and inflammation, angiogenesis, proliferation and apoptosis modulation.

For therapeutic approach an important aspect should be to consider that adiponectin can antagonize the actions of leptin. If adiponectin has been shown to decrease growth and proliferation, increase apoptosis, decrease invasion and vessel density in murine cancer models, leptin has been shown to increase proliferation, migration, and invasion of cancer cells.

Elevated leptin levels have been reported in hepatocellular carcinoma and prostate cancers whereas levels are unchanged in breast cancer patients where leptin receptor expression is instead enhanced. At the same time, adiponectin single-nucleotide polymorphisms have been shown to increase prostate, colon and breast cancer risk. As therapeutic approach recent evidence suggests that soluble leptin receptor can act to bind circulating leptin attenuating its activity, although additional preclinical studies are needed to test the real efficacy *in vivo* [105]. Moreover the use of adiponectin as a direct therapeutic agent is not yet available because of its expensive production and the difficulty in converting the full size adiponectin protein into a drug. Up to date an adiponectin-based short peptide that mimics adiponectin action has been synthesized and called ADP 355 and its test *in vitro* cells reduced the proliferation in a dose-dependent manner [106]. Alternately, targeting downstream adipokine signaling mediators is likely to be a good choice.

**Figure 3:**
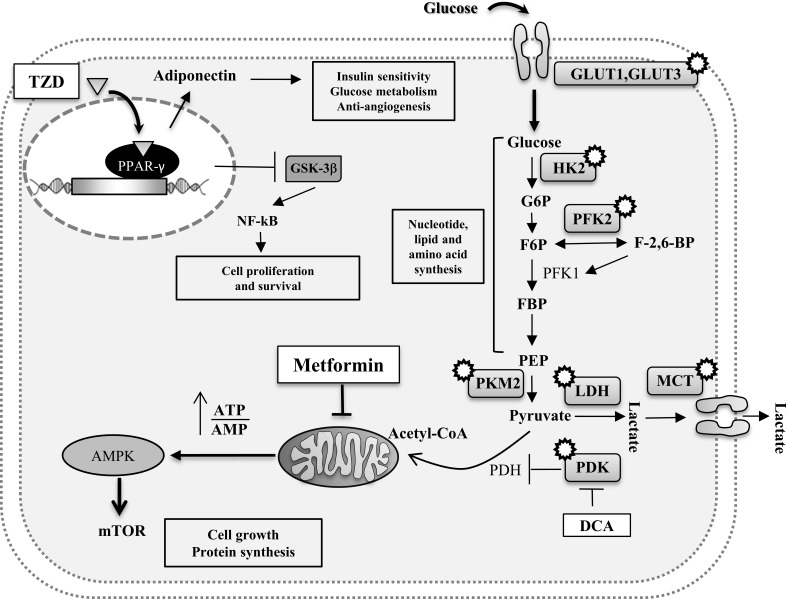
Potential intracellular pathways directly linking MetS with cancer Enzymatic proteins (involved in the Warburg effect) which may represent potential target therapies in oncological patients are also represented (white circles). Drugs are shown in white boxes.

PPAR-γ is highly expressed in adipose tissue and it has high affinity for thiazolidinediones (TZDs) which induces insulin-sensitizing. TZDs which are PPAR-γ agonists increase the secretion of adiponectin from adipocytes altering tumor development but after a long-term treatment. If glitazones (PPARγ agonists) are important to induce antiproliferative or proapoptotic effects in cancer cells taking advantage of the inhibition of glycogen synthase kinase-3β (GSK-3β), a crucial activator of nuclear factor-kappaB (NF-kB), at the same time PPARγ agonists provoke several physiological modifications that influence lipid metabolism, glucose homeostasis and activation of inflammation signaling cascade (Figure 3). It has as consequence that PPARs could have prognostic and/or therapeutic roles but there is urgent need to better understand the real positive effects on tumor treatments. Another controversial aspect of TZDs therapy is that PPAR-γ activation may also affect bone through an increase of bone marrow adiposity and a decrease in osteoblastogenesis, resulting in reduced bone formation [107]. Another down-stream targeting of the adiponectin can be the activation of AMPK through metformin. Metformin inhibits mitochondrial complex I in the liver to interfere with ATP production [108, 109]. This causes an energy stress with the consequent activation of the AMP activated protein (AMPK) *via* an LKB1-dependent mechanism; liver kinase B1 (LKB1) is a protein threonine kinase that has tumor-suppressor activity and it is frequently loss in human cancers (Figure 3). Mechanisms to target the leptin pathway include the use of common inhibitors such as signal transducer and activator of transcription 3 (STAT3), Akt and Raf inhibitors to block cell growth and survival. Dual target therapies directed versus the decreasing response from leptin stimulation and increasing the response from adiponectin pathways have some potential for more efficacious cancer therapy.

Another approach to treat cancer is the one based on targeting the genetic alterations that are known to promote cancer such as the metabolic phenotype that is characterized by cell-autonomous nutrient uptake and reorganization of metabolic pathways to support biosynthesis [110–112]. As described above cancer cells, unlike their normal counterpart, metabolize glucose by aerobic glycolysis. This phenomenon, known as Warburg effect, is characterized by increased glycolysis and lactate production regardless of oxygen availability. It is possible to safely target metabolic pathways in patients. The small molecule dichloroacetate (DCA) is used to treat patients with lactic acidosis resulting from rare inborn errors of mitochondrial metabolism but can be used also to target pyruvate dehydrogenase kinase (PDK). This kinase is expressed in many cancers as a result of increased activation of the transcription factor hypoxia­inducible factor (HIF) [113, 114]. PDK is a negative regulator of the pyruvate dehydrogenase complex (PDH) [115]. PDH catalyses oxidative decarboxylation of pyruvate to acetyl­CoA, which allows the entry of pyruvate into the tricarboxylic acid (TCA) cycle and away from lactate production. Thus, DCA­mediated inhibition of PDK leads to the activation of PDH, increased metabolism of pyruvate to acetyl­CoA and decreased lactate production.

Another therapeutic approach can be to target the glucose transporters which are responsible of glucose uptake. Most of glucose transporters such as GLUT3 and GLUT1 are not expressed in normal cells but they can be expressed at high levels in cancer cells. Antibodies specific for those transporters or analogues which bind the receptor can be a way to block nutrient uptake and starving cancer cells. Some enzymes which are involved in glucose metabolism can be used as therapeutic targets: the hexokinase 2 (HK2) which is responsible of trapping and transforming glucose in glucose 6 phosphate (G6P); the phosphofructokinase 2 (PFK2) which, by generating fructose-2,6-bisphosphate (F-2,6-BP), activates phosphofructokinase 1 (PFK1) to increase flux versus glycolysis; the pyruvate kinase M (PKM2) which promotes aerobic glycolysis etc. but all these enzymes are not so selective for tumoral cells and for most of them the anticancer agent developed is still of limited efficacy for the low tolerability in patients. Because lactate is excreted from the cell, inhibiting lactate production or lactate transport out of the cell are two strategies that directly target the Warburg effect in cancer. The family of monocarboxylate transporters (MCTs) comprises the major proteins that are responsible for lactate export in glycolytic cells, including cancer cells (Figure 3). Considering that the target of MCTs by small molecules also inhibits the proliferation of lymphocytes, this suggests that impaired immune function is a side effect of targeting lactate export in cancer [116].

## CONCLUSIONS AND FUTURE DIRECTIONS

Worldwide, the prevalence of MetS is increasing, and in the United States, nearly two-thirds of adults are either overweight or obese. Given the rising epidemic of metabolic syndrome worldwide, especially in developing countries, and the potential links among MetS, obesity, androgen metabolism, diabetes, and inflammation, it is critical to better understand the complex relations between MetS and cancer risk and the role of chronic inflammation in MetS and the pathogenesis of cancer.

Clearly, to dissect these interrelated factors, future prospective studies should be sufficiently large, with better assessment of overall and abdominal obesity and with biochemical measures, such as insulin concentrations, sex steroids, and IGFs, to clarify the complex interplays of these factors on cancer risk. Etiologic heterogeneity should be considered. Further refinement of molecular cancer classification, using biomarkers and genetic markers, coupled with a clearer understanding of the cellular and molecular pathways involved, should prove illuminating. Factors such as grade, stage, and aggressiveness of tumors should be assessed and incorporated into the analysis. Methodological studies are also needed to gain a better understanding of the determinants of these biomarkers, including insulin, leptin, adipokines, IGFs, sex steroids, and inflammatory mediators, and to provide biological data to help interpret the results.

A potential role for IGF-IR target therapy, PPARs agonists, TZDs and metformin in the adjuvant treatment of cancers is advisable, but further studies are warranted in order to better clarify the impact of these drugs in cancer therapy. At the time of writing, nearly 60 patents have been filed for small-molecule activators of AMPK, and it is hoped that some of these may enter human clinical trials soon. It seems likely that by the end of this decade we will have a much clearer picture of whether drugs that are selective for MetS will have a place in the treatment of cancer.

## References

[1] Nashar K, Egan BM (2014). Relationship between chronic kidney disease and metabolic syndrome: current perspectives. Diabetes Metab Syndr Obes.

[2] Athyros VG, Ganotakis ES, Tziomalos K, Papageorgiou AA, Anagnostis P, Griva T, Kargiotis K, Mitsiou E K, Karagiannis A, Mikhailidis DP (2010). Comparison of four definitions of the metabolic syndrome in a Greek (Mediterranean) population. Curr Med Res Opin.

[3] Alberti KG, Eckel RH, Grundy SM, Zimmet PZ, Cleeman JI, Donato KA, Fruchart JC, James WP, Loria CM, Smith SC (2009). Harmonizing the metabolic syndrome: a joint interim statement of the International Diabetes Federation Task Force on Epidemiology and Prevention; National Heart, Lung, and Blood Institute; American Heart Association; World Heart Federation; International Atherosclerosis Society; and International Association for the Study of Obesity. Circulation.

[4] Ford ES, Giles WH, Dietz WH (2002). Prevalence of the metabolic syndrome among US adults: findings from the third National Health and Nutrition Examination Survey. JAMA.

[5] Cameron AJ, Shaw JE, Zimmet PZ (2006). The metabolic syndrome: prevalence in worldwide populations. Endocrinol Metab Clin North Am.

[6] Ford ES, Giles WH, Mokdad AH (2004). Increasing prevalence of the metabolic syndrome among U.S. adults. Diabetes Care.

[7] Esposito K, Chiodini P, Colao A, Lenzi A, Giugliano D (2012). Metabolic syndrome and risk of cancer. Diabetes Care.

[8] Giuliano D, Ceriello A, Esposito K (2008). Are there specific treatments for the metabolic syndrome?. Am J Clin Nutr.

[9] Giovannucci E, Harlan DM, Archer MC, Bergenstal RM, Gapstur SM, Habel LA, Pollak M, Regensteiner JG, Yee D (2010). Diabetes and cancer: a consensus report. Diabetes Care.

[10] Jaggers JR, Sui X, Hooker SP, LaMonte MJ, Matthews CE, Hand GA, Blair SN (2009). Metabolic syndrome and risk of cancer mortality in men. Eur J Cancer.

[11] Stebbing J, Sharma A, North B, Athersuch TJ, Zebrowski A, Pchejetski D, Coombes RC, Nicholson JK, Keun HC (2012). A metabolic phenotyping approach to understanding relationships between metabolic syndrome and breast tumour responses to chemotherapy. Ann Oncol.

[12] Hoffman KE, Derdak J, Bernstein D, Reynolds JC, Avila NA, Gerber L, Steinberg SM, Chrousos G, Mackall CL, Mansky PJ (2008). Metabolic syndrome traits in long-term survivors of pediatric sarcoma. Pediatr Blood Cancer.

[13] Nuver J, Smit AJ, Wolffenbuttel BH, Sluiter WJ, Hoekstra HJ, Sleijfer DT, Gietema JA (2005). The metabolic syndrome and disturbances in hormone levels in long-term survivors of disseminated testicular cancer. J Clin Oncol.

[14] Braga-Basaria M, Dobs AS, Muller DC, Carducci MA, Jhon M, Egan J, Basaria S (2006). Metabolic syndrome in men with prostate cancer undergoing long-term androgen-deprivation therapy. J Clin Oncol.

[15] de Haas EC, Oosting SF, Lefrandt JD, Wolffenbuttel BH, Sleijfer DT, Gietema JA (2010). The metabolic syndrome in cancer survivors. Lancet Oncol.

[16] James WTP, Rigby N, Leach R (2004). The obesity epidemic, metabolic syndrome and future prevention strategies. Eur J Cardiovasc Prev Rehabil.

[17] Key TJ, Spencer EA, Reeves GK (2010). Symposium 1: overnutrition: consequences and solutions. Obesity and cancer risk. Proc Nutr Soc.

[18] Pan SY, Johnson KC, Ugnat AM, Wen SW, Mao Y (2004). Canadian Cancer Registries Epidemiology Research Group. Association of obesity and cancer risk in Canada. Am J Epidemiol.

[19] Renehan AG, Tyson M, Egger M, Heller RF, Zwahlen M (2008). Body-mass index and incidence of cancer: a systematic review and meta-analysis of prospective observational studies. Lancet.

[20] Calle EE, Rodriguez C, Walker-Thurmond K, Thun MJ (2003). Overweight, obesity, and mortality from cancer in a prospectively studied cohort of U.S. adults. N Engl J Med.

[21] Jemal A, Siegel R, Xu J, Ward E (2010). Cancer statistics. CA Cancer J Clin.

[22] Wellen KE, Hotamisligil GS (2003). Obesity-induced inflammatory changes in adipose tissue. J Clin Invest.

[23] Trayhurn P, Wood IS (2004). Adipokines: inflammation and the pleiotropic role of white adipose tissue. Br J Nutr.

[24] Cowey S, Hardy RW (2006). The metabolic syndrome: A high-risk state for cancer?. Am J Pathol.

[25] Piatkiewicz P, Czech A (2011). Glucose metabolism disorders and the risk of cancer. Arch Immunol Ther Exp.

[26] Bråkenhielm E, Veitonmäki N, Cao R, Kihara S, Matsuzawa Y, Zhivotovsky B, Funahashi T, Cao Y (2004). Aiponectin-induced antiangiogenesis and antitumor activity involved caspase-mediated endothelial cell apoptosis. Proc Natl Acad Sci USA.

[27] Ishikawa M, Kitayama J, Kazama S, Hiramatsu T, Hatano K, Nagawa H (2005). Plasma adiponectin and gastric cancer. Clin Cancer Res.

[28] Górska E, Popko K, Winiarska M, Wasik M (2009). (Pleiotropic effects of leptin) (in Polish). Pediatr Endocrinol Diabetes Metab.

[29] Houseknecht KL, Baile CA, Matteri RL, Spurlock ME (1998). The biology of leptin: a review. J Anim Sci.

[30] Garofalo C, Surmacz E (2006). Leptin and cancer. J Cell Physiol.

[31] Silha JV, Krsek M, Sucharda P, Murphy LJ (2005). Angiogenic factors are elevated in overweight and obese individuals. Int J Obes (Lond).

[32] Miyazawa-Hoshimoto S, Takahashi K, Bujo H, Hashimoto N, Saito Y (2003). Elevated serum vascular endothelial growth factor is associated with visceral fat accumulation in human obese subjects. Diabetologia.

[33] Kolonin MG, Simmons PJ (2009). Combinatorial stem cell mobilization. Nat Biotechnol.

[34] Bianco P, Robey PG, Simmons PJ (2008). Mesenchymal stem cells: revisiting history, concepts, and assays. Cell Stem Cell.

[35] Pittenger MF, Mackay AM, Beck SC, Jaiswal RK, Douglas R, Mosca JD, Moorman MA, Simonetti DW, Craig S, Marshak DR (1999). Multilineage potential of adult human mesenchymal stem cells. Science.

[36] Prockop DJ (1997). Marrow stromal cells as stem cells for non-hematopoietic tissues. Science.

[37] Eterno V, Zambelli A, Pavesi L, Villani L, Zanini V, Petrolo G, Manera S, Tuscano A, Amato A (2014). Adipose-derived mesenchymal stem cells (ASCs) may favour breast cancer recurrence *via* HGF/c-Met signaling. Oncotarget.

[38] Duvnjak L, Duvnjak M (2009). The metabolic syndrome - an ongoing story. J Physiol Pharmacol.

[39] Bergamini E, Cavallini G, Donati A, Gori Z (2007). The role of autophagy in aging: its essential part in the anti-aging mechanism of caloric restriction. Ann N Y Acad Sci.

[40] Sieradzki J, Szczeklik A (2005). Cukrzyca i zespół metaboliczny. Choroby Wewnętrzne.

[41] Djiogue S, Nwabo Kamdje AH, Vecchio L, Kipanyula MJ, Farahna M, Aldebasi Y, Seke Etet PF (2013). Insulin resistance and cancer: the role of insulin and IGFs. Endocr Relat Cancer.

[42] Sakurai T, Kudo M (2011). Signaling pathways governing tumor angiogenesis. Oncology.

[43] Pollak M (2012). The insulin and insulin-like growth factor receptor family in neoplasia: an update. Nat Rev Cancer.

[44] Seke Etet PF, Vecchio L, Nwabo Kamdje AH (2012). Interactions between bone marrow stromal microenvironment and B-chronic lymphocytic leukemia cells: any role for Notch, Wnt and Hh signaling pathways?. Cell Signal.

[45] Sun Ha J, Hee JK, Jakyoung L (2005). Obesity, Insulin Resistance and Cancer Risk. Yonsei Med J.

[46] Calle EE, Kaaks R (2004). Overweight, obesity and cancer: epidemiological evidence and proposed mechanisms. Nat Rev Cancer.

[47] Vander Heiden MG, Cantley LC, Thompson CB (2009). Understanding the Warburg effect: the metabolic requirements of cell proliferation. Science.

[48] Airley RE, Mobasheri A (2007). Hypoxic regulation of glucose transport, anaerobic metabolism and angiogenesis in cancer: novel pathways and targets for anticancer therapeutics. Chemotherapy.

[49] Macheda ML, Rogers S, Best JD (2005). Molecular and cellular regulation of glucose transporter (GLUT) proteins in cancer. J Cell Physiol.

[50] Yan H, Parsons DW, Jin G, McLendon R, Rasheed BA, Yuan W, Kos I, Batinic-Haberle I, Jones S, Riggins GJ, Friedman H, Friedman A, Reardon D (2009). IDH1 and IDH2 mutations in gliomas. N Engl J Med.

[51] King A, Selak MA, Gottlieb E (2006). Succinate dehydrogenase and fumarate hydratase: linking mitochondrial dysfunction and cancer. Oncogene.

[52] Hao HX, Khalimonchuk O, Schraders M, Dephoure N, Bayley JP, Kunst H, Devilee P, Cremers CW, Schiffman JD, Bentz BG, Gygi SP, Winge DR, Kremer H, Rutter J (2009). SDH5, a gene required for flavination of succinate dehydrogenase, is mutated in paraganglioma. Science.

[53] Smith TA (2000). Mammalian hexokinases and their abnormal expression in cancer. Br J Biomed Sci.

[54] Minchenko OH, Ochiai A, Opentanova IL, Ogura T, Minchenko DO, Caro J, Komisarenko SV, Esumi H (2005). Overexpression of 6-phosphofructo-2-kinase/fructose-2,6-bisphosphatase-4 in the human breast and colon malignant tumors. Biochimie.

[55] Stattin P, Björ O, Ferrari P, Lukanova A, Lenner P, Lindahl B, Hallmans G, Kaaks R (2007). Prospective study of hyperglycemia and cancer risk. Diabetes Care.

[56] Micucci C, Orciari S, Catalano A (2014). Hyperglycemia promotes K-Ras-induced lung tumorigenesis through BASCs amplification. PLoS One.

[57] Iwatsuki M, Mimori K, Yokobori T, Ishi H, Beppu T, Nakamori S, Baba H, Mori M (2010). Epithelial-mesenchymal transition in cancer development and its clinical significance. Cancer Sci.

[58] Dong C, Yuan T, Wu Y, Wang Y, Fan TW, Miriyala S, Lin Y, Yao J, Shi J, Kang T, Lorkiewicz P, St Clair D, Hung MC, Evers BM, Zhou BP (2013). Loss of FBP1 by Snail-mediated repression provides metabolic advantages in basal-like breast cancer. Cancer Cell.

[59] Masur K, Vetter C, Hinz A, Tomas N, Henrich H, Niggemann B, Zänker KS (2011). Diabetogenic glucose and insulin concentrations modulate transcriptome and protein levels involved in tumour cell migration, adhesion and proliferation. Br J Cancer.

[60] LeRoith D, Roberts CT (2003). The insulin-like growth factor system and cancer. Cancer Lett.

[61] Khandwala HM, McCutcheon IE, Flyvbjerg A, Friend KE (2000). The effects of insulin-like growth factors on tumorigenesis and neoplastic growth. Endocr Rev.

[62] Frasca F, Pandini G, Vigneri R, Goldfine ID (2003). Insulin and hybrid insulin/IGF receptors are major regulators of breast cancer cells. Breast Dis.

[63] Hankinson SE, Willett WC, Colditz GA, Hunter DJ, Michaud DS, Deroo B, Rosner B, Speizer FE, Pollak M (1998). Circulating concentrations of insulin-like growth factor-I and risk of breast cancer. Lancet.

[64] Chan JM, Stampfer MJ, Giovannucci E, Gann PH, Ma J, Wilkinson P, Hennekens CH, Pollak M (1998). Plasma insulin-like growth factor-I and prostate cancer risk: a prospective study. Science.

[65] Yu H, Spitz MR, Mistry J, Gu J, Hong WK, Wu X (1999). Plasma levels of insulin-like growth factor-I and lung cancer risk: a case-control study. J Natl Cancer Inst.

[66] Ma J, Pollak MN, Giovannucci E, Chan JM, Tao Y, Hennekens CH, Stampfer MJ (1999). Prospective study of colorectal cancer risk in men and plasma levels of insulin-like growth factor (IGF)-I and IGF-binding protein-3. J Natl Cancer Inst.

[67] Price AJ, Allen NE, Appleby PN, Crowe FL, Travis RC, Tipper SJ, Overvad K, Grønbæk H, Tjønneland A, Johnsen NF, Rinaldi S, Kaaks R, Lukanova A (2012). Insulin-like growth factor-I concentration and risk of prostate cancer: results from the European Prospective Investigation into Cancer and Nutrition. Cancer Epidemiol Biomarkers Prev.

[68] Koutsilieris M, Frenette G, Lazure C, Lehoux JG, Govindan MV, Polychronakos C (1993). Urokinase-type plasminogen activator: a paracrine factor regulating the bioavailability of IGFs in PA-III cell-induced osteoblastic metastases. Anticancer Research.

[69] Culig Z, Hobisch A, Cronauer MV, Radmayr C, Trapman J, Hittmair A, Bartsch G, Klocker H (1994). Androgen receptor activation in prostatic tumor cell lines by insulin-like growth factor-I, keratinocyte growth factor, and epidermal growth factor. Cancer Research.

[70] Rabiau N, Dechelotte P, Adjakly M, Kemeny JL, Guy L, Boiteux JP, Kwiatkowski F, Bignon YJ, Bernard-Gallon D (2011). BRCA1, BRCA2, AR and IGF-I expression in prostate cancer: correlation between RT-qPCR and immunohistochemical detection. Oncology Reports.

[71] Keku TO, Lund PK, Galanko J, Simmons JG, Woosley JT, Sandler RS (2005). Insulin resistance, apoptosis, and colorectal adenoma risk. Cancer Epidemiol Biomarkers Prev.

[72] Kukliński A, Kamocki Z, Cepowicz D, Gryko M, Czyżewska J, Pawlak K, Kędra B (2011). Relationships between insulin-like growth factor I and selected clinico-morphological parameters in colorectal cancer patients. Pol Przegl Chir.

[73] Rosen N, Yee D, Lippman ME, Paik S, Cullen KJ (1991). Insulin-like growth factors in human breast cancer. Breast Cancer Res Treat.

[74] Bruning PF, Van Doorn J, Bonfrèr JM, Van Noord PA, Korse CM, Linders TC, Hart AA (1995). Insulin-like growth-factor-binding protein 3 is decreased in early-stage operable pre-menopausal breast cancer. Int J Cancer.

[75] Pasanisi P, Bruno E, Venturelli E, Manoukian S, Barile M, Peissel B, De Giacomi C, Bonanni B, Berrino J, Berrino F (2011). Serum levels of IGF-I and BRCA penetrance: a case control study in breast cancer families. Fam. Cancer.

[76] Al-Delaimy WK, Flatt SW, Natarajan L, Laughlin GA, Rock CL, Gold EB, Caan BJ, Parker BA, Pierce JP (2011). IGF1 and risk of additional breast cancer in the WHEL study. Endocr Relat Cancer.

[77] Jiang Y, Wang L, Gong W, Wei D, Le X, Yao J, Ajani J, Abbruzzese JL, Huang S, Xie K (2004). A high expression level of insulin-like growth factor I receptor is associated with increased expression of transcription factor Sp1 and regional lymph node metastasis of human gastric cancer. Clin Exp Metastasis.

[78] Parker A, Cheville JC, Lohse C, Cerhan JR, Blute ML (2003). Expression of insulin-like growth factor I receptor and survival in patients with clear cell renal cell carcinoma. J Urol.

[79] Humpert PM, Djuric Z, Zeuge U, Oikonomou D, Seregin Y, Laine K, Eckstein V, Nawroth PP, Bierhaus A (2008). Insulin stimulates the clonogenic potential of angiogenic endothelial progenitor cells by IGF-1 receptor-dependent signaling. Mol Med.

[80] Asahara T, Murohara T, Sullivan A, Silver M, van der Zee R, Li T, Witzenbichler B, Schatteman G, Isner JM (1997). Isolation of putative progenitor endothelial cells for angiogenesis. Science.

[81] Shaked Y, Ciarrocchi A, Franco M, Lee CR, Man S, Cheung AM, Hicklin DJ, Chaplin D, Foster FS, Benezra R, Kerbel RS (2006). Therapy-induced acute recruitment of circulating endothelial progenitor cells to tumors. Science.

[82] Valko M, Izakovic M, Mazur M, Rhodes CJ, Telser J (2004). Role of oxygen radicals in DNA damage and cancer incidence. Mol Cell Biochem.

[83] Howe LR, Subbaramaiah K, Hudis CA, Dannenberg AJ (2013). Molecular pathways: adipose inflammation as a mediator of obesity-associated cancer. Clin. Cancer Res.

[84] Hursting SD, Digiovanni J, Dannenberg AJ, Azrad M, Leroith D, Demark-Wahnefried W, Kakarala M, Brodie A, Berger NA (2012). Obesity, energy balance, and cancer: new opportunities for prevention. Cancer Prev Res.

[85] Braun S, Bitton-Worms K, LeRoith D (2011). The link between the metabolic syndrome and cancer. Int J Biol Sci.

[86] Iyengar NM, Hudis CA, Dannenberg AJ (2015). Obesity and cancer: local and systemic mechanisms. Annu Rev Med.

[87] Cinti S, Mitchell G, Barbatelli G, Murano I, Ceresi E, Faloia E, Wang S, Fortier M, Greenberg AS, Obin MS (2005). Adipocyte death defines macrophage localization and function in adipose tissue of obese mice and humans. J Lipid Res.

[88] Vidyasagar S, Abdul Razak UK, Prashanth CK, Muralidhar Varma D, Bairy KL (2013). Highly sensitive C-reactive protein in metabolic syndrome. JIACM.

[89] Erlinger TP, Platz EA, Rifai N, Helzlsouer KJ (2004). C-reactive protein and the risk of incident colorectal cancer. JAMA.

[90] Key TJ, Appleby PN, Reeves GK, Roddam A, Dorgan JF, Longcope C, Stanczyk FZ, Stephenson HE, Falk RT, Miller R, Schatzkin A, Allen DS, Fentiman IS, Key TJ (2003). Endogenous Hormones Breast Cancer Collaborative Group. Body mass index, serum sex hormones, and breast cancer risk in postmenopausal women. J Natl Cancer Inst.

[91] Pugeat M, Crave JC, Elmidani M, Nicolas MH, Garoscio-Cholet M, Lejeune H, Déchaud H, Tourniaire J (1991). Pathophysiology of sex hormone binding globulin (SHBG): relation to insulin. J Steroid Biochem Mol Biol.

[92] Dickson RB, Stancel GM (2000). Estrogen receptor-mediated processes in normal and cancer cells. J Natl Cancer Inst. Monogr.

[93] Flötotto T, Djahansouzi S, Gläser M, Hanstein B, Niederacher D, Brumm C, Beckmann MW (2001). Hormones and hormone antagonists: mechanisms of action in carcinogenesis of endometrial and breast cancer. Horm Metab Res.

[94] Key TJ, Pike MC (1988). The dose-effect relationship between ‘unopposed’ estrogens and endometrial mitotic rate: its central role in explaining and predicting endometrial cancer. Br J Cancer.

[95] Yager JD, Davidson NE (2006). Estrogen carcinogenesis in breast cancer. N Engl J Med.

[96] Péqueux C, Raymond-Letron I, Blacher S, Boudou F, Adlanmerini M, Fouque MJ, Rochaix P, Noël A, Foidart JM, Krust A, Chambon P, Brouchet L, Arnal JF, Lenfant F (2012). Stromal estrogen receptor-α promotes tumor growth by normalizing an increased angiogenesis. Cancer Res.

[97] Key T, Appleby P, Barnes I, Reeves G (2002). Endogenous Hormones and Breast Cancer Collaborative Group. Endogenous sex hormones and breast cancer in postmenopausal women: reanalysis of nine prospective studies. J Natl Cancer Inst.

[98] Zeleniuch-Jacquotte A, Shore RE, Koenig KL, Akhmedkhanov A, Afanasyeva Y, Kato I, Kim MY, Rinaldi S, Kaaks R, Toniolo P (2004). Postmenopausal levels of oestrogen, androgen, and SHBG and breast cancer: long-term results of a prospective study. Br J Cancer.

[99] Lukanova A, Lundin E, Micheli A, Arslan A, Ferrari P, Rinaldi S, Krogh V, Lenner P, Shore RE, Biessy C, Muti P, Riboli E, Koenig KL (2004). Circulating levels of sex steroid hormones and risk of endometrial cancer in postmenopausal women. Int J Cancer.

[100] Zeleniuch-Jacquotte A, Akhmedkhanov A, Kato I, Koenig KL, Shore RE, Kim MY, Levitz M, Mittal KR, Raju U, Banerjee S, Toniolo P (2001). Postmenopausal endogenous oestrogens and risk of endometrial cancer: results of a prospective study. Br J Cancer.

[101] Moschos SJ, Mantzoros CS (2002). The role of the IGF system in cancer: from basic to clinical studies and clinical applications. Oncology.

[102] Yee D, Paik S, Lebovic GS, Marcus RR, Favoni RE, Cullen KJ, Lippman ME, Rosen N (1989). Analysis of insulin-like growth factor I gene expression in malignancy: evidence for a paracrine role in human breast cancer. Mol Endocrinol.

[103] Chen HX, Sharon E (2013). IGF-1R as an anti-cancer target - trials and tribulations. Chin J Cancer.

[104] Janssen JA, Varewijck AJ (2014). IGF IR Targeted Therapy: Past, Present and Future. Front Endocrinol. (Lausanne).

[105] Dalamaga M, Diakopoulos KN, Mantzoros CS (2012). The role of adiponectin in cancer: a review of current evidence. Endocr Rev.

[106] Otvos L, Haspinger E, La Russa F, Maspero F, Graziano P, Kovalszky I, Lovas S, Nama K, Hoffmann R, Knappe D, Cassone M, Wade J, Surmacz E. (2011). Design and development of a peptide-based adiponectin receptor agonist for cancer treatment. BMC Biotechnol.

[107] Rzonca SO, Suva LJ, Gaddy D, Montague DC, Lecka-Czernik B (2004). Bone is a target for the antidiabetic compound rosiglitazone. Endocrinology.

[108] El-Mir MY, Nogueira V, Fontaine E, Avéret N, Rigoulet M, Leverve X (2000). Dimethylbiguanide inhibits cell respiration *via* an indirect effect targeted on the respiratory chain complex I. J Biol Chem.

[109] Buzzai M, Jones RG, Amaravadi RK, Lum JJ, DeBerardinis RJ, Zhao F, Viollet B, Thompson CB (2007). Systemic treatment with the antidiabetic drug metformin selectively impairs p53-deficient tumor cell growth. Cancer Res.

[110] Deberardinis RJ, Lum JJ, Hatzivassiliou G, Thompson CB (2008). The biology of cancer: metabolic reprogramming fuels cell growth and proliferation. Cell Metab.

[111] Cairns RA, Harris IS, Mak TW (2011). Regulation of cancer cell metabolism. Nature Rev Cancer.

[112] Locasale JW, Cantley LC, Vander Heiden MG (2009). Cancer's insatiable appetite. Nature Biotech.

[113] Kim JW, Tchernyshyov I, Semenza GL, Dang CV (2006). HIF 1 mediated expression of pyruvate dehydrogenase kinase: a metabolic switch required for cellular adaptation to hypoxia. Cell Metab.

[114] Papandreou I, Cairns RA, Fontana L, Lim AL, Denko NC (2006). HIF 1 mediates adaptation to hypoxia by actively downregulating mitochondrial oxygen consumption. Cell Metab.

[115] Holness MJ, Sugden MC (2003). Regulation of pyruvate dehydrogenase complex activity by reversible phosphorylation. Biochem Soc Trans.

[116] Vander Heiden MG (2011). Targeting cancer metabolism: a therapeutic window opens. Nat Rev Drug Discov.

